# Prefrontal brain stimulation during food-related inhibition training: effects on food craving, food consumption and inhibitory control

**DOI:** 10.1098/rsos.181186

**Published:** 2019-01-09

**Authors:** Jemma Sedgmond, Natalia S. Lawrence, Frederick Verbruggen, Sinead Morrison, Christopher D. Chambers, Rachel C. Adams

**Affiliations:** 1 Cardiff University Brain Research Imaging Centre (CUBRIC), School of Psychology, Cardiff University, Cardiff CF24 4HQ, UK; 2 School of Psychology, University of Exeter, Washington Singer Building, Exeter EX4 4QG, UK; 3 Department of Experimental Psychology, Ghent University, Henri Dunantlaan 2, 9000 Ghent, Belgium

**Keywords:** transcranial direct current stimulation, brain stimulation, food intake, food craving, inhibitory control

## Abstract

Modulation of dorsolateral prefrontal cortex (DLPFC) activity using non-invasive brain stimulation has been shown to reduce food craving as well as food consumption. Using a preregistered design, we examined whether bilateral transcranial direct current stimulation (tDCS) of the DLPFC could reduce food craving and consumption in healthy participants when administered alongside the cognitive target of inhibitory control training. Participants (*N* = 172) received either active or sham tDCS (2 mA; anode F4, cathode F3) while completing a food-related Go/No-Go task. State food craving, ad-lib food consumption and response inhibition were evaluated. Compared with sham stimulation, we found no evidence for an effect of active tDCS on any of these outcome measures in a predominantly female sample. Our findings raise doubts about the effectiveness of single-session tDCS on food craving and consumption. Consideration of individual differences, improvements in tDCS protocols and multi-session testing are discussed.

## 1. Introduction

Unprocessed or minimally processed foods account for less than 30% of the average UK diet, with ultra-processed foods contributing more than 50% of food intake [1]. The availability and low cost of highly palatable and energy-dense food has resulted in increased consumption [2,3], providing one of the leading explanations for rising rates of obesity [2,4]. It has now been noted that this rise in food consumption is often in the absence of hunger and is instead driven by factors unrelated to an individual's physiological requirements, such as pleasure [5]. This desire to consume food is referred to as hedonic hunger, and is satisfied by the consumption of these energy- and calorie-dense foods [5]. Hedonic hunger can manifest as a preoccupation with constant thoughts surrounding food, resulting in persistent cravings [6]. It is these cravings—which manifest as a strong desire to consume a specific food—that can result in the overconsumption of food regardless of caloric requirement [7]. However, the extent to which hedonic factors influence eating behaviour varies widely across individuals; for some, healthy eating remains a constant challenge, whereas others appear able to resist temptation and maintain a healthy diet.

Neuroimaging studies have suggested that individual differences in prefrontal brain activity related to goal-oriented behaviour and self-control may help to explain vulnerability to hedonic eating [8–10]. A particular region of interest is the dorsolateral prefrontal cortex (DLPFC) which has been associated with the impulsiveness often linked to overconsumption [11]. In a meta-analysis, Brooks *et al.* [12] found that obese participants showed reduced activation of the left DLPFC in response to food images. Similarly, those who are obese have been shown to have reduced grey matter volume in the left inferior frontal gyrus and bilateral DLPFC [13]. Increased activity in the DLPFC, on the other hand, has been associated with successful self-regulation of food intake and weight loss [14–16]. It has therefore been proposed that enhancing brain activity within the DLPFC may help to increase self-control and reduce food consumption [17].

Transcranial direct current stimulation (tDCS) has frequently been used in studies of food-related behaviour with the aim of modifying automatic responses to food stimuli. tDCS involves the delivery of a weak (typically 1–2 mA) direct electrical current to the cortex via two scalp electrodes. The effect of tDCS on brain activity is dependent on the stimulation polarity; anodal stimulation is thought to increase cortical excitability by neuronal depolarization, whereas cathodal stimulation is believed to decrease excitability by hyperpolarizing neurons [18–23].

Activation of the DLPFC using tDCS has indeed shown promise for modulating food-related behaviour. Compared to sham stimulation, active tDCS has been found to reduce food craving in healthy subjects who self-identify as having frequent and strong cravings [24–28]. Furthermore, it seems that the effect of tDCS goes beyond craving alone and can result in decreased food consumption [24,27,29]. For example, Fregni *et al*. [24] investigated the potential effects of tDCS on both food craving and food consumption using a within-subjects crossover design. Participants' craving scores were measured before and after exposure to nine processed food items while watching a 5-min film depicting images of foods known to elicit cravings. These measures were then repeated following sham or active DLPFC stimulation with an ad-libitum eating phase post-exposure. A significant reduction in food craving was found in the anode right/cathode left condition, though not in the anode left/cathode right condition, and sham stimulation resulted in a significant increase in food craving. Overall calorie consumption was also significantly lower in both active conditions compared to sham, with the anode right/cathode left condition resulting in the lowest intake. This finding was replicated by Goldman *et al*. [25], who also demonstrated a significantly greater reduction in both food craving and an inability to resist food with active anodal right/ cathodal left stimulation compared to sham stimulation, although they found no difference in food consumption. More recent studies have also replicated effects of tDCS on food craving, although effects on food consumption are more equivocal [26,27]. Potential explanations for variability in outcomes are inadequate statistical power (the largest sample size across these papers was *N* = 21; see [30]) and suboptimal study protocols, including lack of study preregistration to control various forms of analytic and reporting bias.

Unlike some forms of brain stimulation, tDCS is a subthreshold intervention; it is too weak to induce activity, but instead modulates already occurring neuronal activity [31]. It has been argued, therefore, that the effectiveness of tDCS may be improved with the addition of a cognitive task that promotes activity in the target brain regions [17,18,31,32]. One potential task that could augment the effect of tDCS on food cravings and consumption is food-related inhibition training.

Food-related inhibition training typically requires participants to withhold their responses to images of palatable foods in response inhibition tasks such as the Stop-Signal task or Go/No-Go task. Previous studies have suggested that such training may be effective in modifying food-related behaviour and can result in decreased consumption of unhealthy foods, healthier food choices, and even weight loss [33–38]. Two recent meta-analyses have reported small but significant effect sizes for the effect of inhibition training on food consumption and have further indicated that effects are greater for Go/No-Go compared to Stop-Signal training ([39,40]; see also [33]). Furthermore, inhibitory control (especially in the Go-No-Go task) has been linked to activation within the DLPFC [41–47]. For example, using electroencephalography (EEG), Lapenta *et al*. [27] found that bilateral tDCS to the DLPFC (anodal right/ cathodal left) resulted in reduced N2 and increased P3a components of responses to No-Go stimuli. The authors also found reduced food craving and consumption following active stimulation and suggested that these effects were mediated by changes in inhibitory control. Although no studies to date have paired food-related inhibition training with DLPFC stimulation, one recent study has combined general inhibition training with stimulation of the inferior frontal gyrus—another area believed to be involved in response inhibition [48]. Ditye *et al*. found that the combination of training and stimulation was more effective at improving performance than just inhibition training alone. However, because the training-only group did not receive sham stimulation it is possible that these results were due to non-specific effects of brain stimulation, including a placebo effect.

The present study therefore aimed to extend previous findings by investigating whether combining tDCS and food-related inhibition training could have a cumulative effect on decreasing food cravings and consumption. Furthermore, we recruited a larger-than-typical sample size (*N* = 172) and all methods were pre-registered prior to data acquisition to ensure transparency and reduce researcher bias. Using a between-subjects design, participants were randomly assigned to receive either active or sham stimulation; in accordance with previous studies we delivered bilateral, anodal right/cathodal left DLPFC stimulation (e.g. [24]). Stimulation was paired with a food-related Go/No-Go training task in which unhealthy foods were consistently paired with inhibition and healthy foods were paired with a response. State food cravings were measured before and after tDCS, and following stimulation participants were presented with a snack buffet to measure ad-libitum food consumption. The snack buffet contained the same foods presented during training in addition to two novel foods (one unhealthy and one healthy). To justify the snack phase participants were informed that we were measuring the effect of blood glucose levels on cognitive performance. We therefore needed to measure performance at the beginning of the study, following a three hour fast, and following food intake. Our primary pre-registered hypothesis was that participants receiving active tDCS would consume fewer calories than those receiving sham tDCS, with a secondary pre-registered hypothesis that the active group would also show a decrease in food craving compared with sham. A speeded Go/No-Go task was also included at the end of the session as a pre-registered manipulation check for the effect of tDCS on inhibitory control; we predicted that participants in the active group would make fewer commission errors compared with the sham group (the percentage of erroneous responses made on no-go trials).

## 2. Method

### 2.1. Participants and sample size

One hundred and eighty-one participants (141 females, age: *M* = 20.81, s.e. = 0.26) were recruited from the staff and student population at Cardiff University and 172 participants were included in the final analyses following exclusions according to pre-registered criteria (134 females, age: *M* = 20.8, s.e. = 0.26). Participants were all aged 18–45, right-handed, and had no contraindications for tDCS. Participants were excluded if they were currently dieting (with the aim to lose weight), if they had any history of eating disorders or if they had previously taken part in any studies involving inhibition training and food consumption. Sample size was determined according to an *a priori* power calculation. Although we used a Bayesian inferential stopping rule for the main effect of total calorie intake between groups, we achieved our maximum possible sample size before the Bayes factor reached the recommended threshold for early research (BF > 6 or BF < 1/6 to provide moderate evidence for the experimental or null hypothesis, respectively; [51]; see electronic supplementary material for further details). Our maximum sample size of 172 participants provided us with 90% power to detect an effect size of *d* = 0.5 using a two-tailed independent *t*-test with an alpha level of 0.05 (G*Power; [52]). All participants were reimbursed at a rate of £10 per hour. The study was approved by the School of Psychology Research Ethics Committee, Cardiff University.

### 2.2. Materials/measures

#### 2.2.1. Transcranial direct current stimulation

Participants received either active or sham tDCS. Two 7 × 5 cm (35 cm^2^), saline-soaked, sponge electrodes were positioned bilaterally with the anode placed over the right DLPFC and the cathode over the left DLPFC (F4 and F3 respectively using the 10–20 EEG system). For the active condition a 2 mA current was applied using a battery-driven constant-current stimulator (Neuroconn DC-STIMULATOR PLUS, neuroConn GmbH, Illmenau, Germany) for 20 min (with a 10 s ramp up and down). For the sham condition, the stimulator delivered a 2 mA current for 30 s before being ramped down to 0 mA over a 1 min period. The experimenter was provided with a study code for each participant that would generate either active or sham stimulation, ensuring that the experimenter was blinded to the condition.

#### 2.2.2. Go/No-Go training task

**Figure 1.**
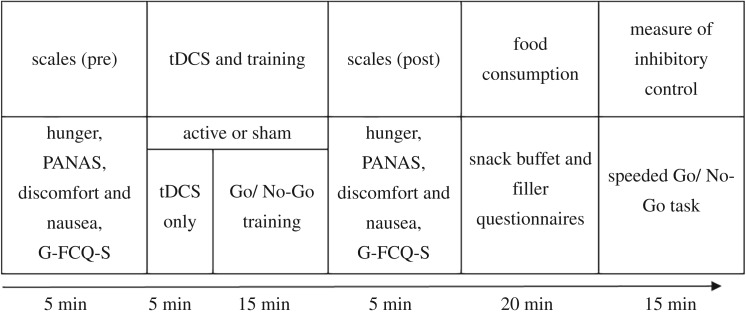
Schematic diagram of the procedure. Participants initially completed measures of hunger, mood and craving before being randomly allocated to receive either active or sham stimulation. After 5 min of stimulation a food-related Go/No-Go training task was introduced. Following this task, participants were presented with the hunger, mood and craving scales and then a snack buffet with various unhealthy and healthy foods for consumption. Filler questionnaires were provided during the buffet to keep participants occupied for 20 min. Participants then completed a speeded version of the Go/No-Go task to measure inhibitory control (full details can be found in the Method section). *Note*. PANAS = Positive and Negative Affect Schedule [49]; G-FCQ-S = General Food Craving Questionnaire – State Version [50]; tDCS = transcranial direct current stimulation.

**Figure 2.**
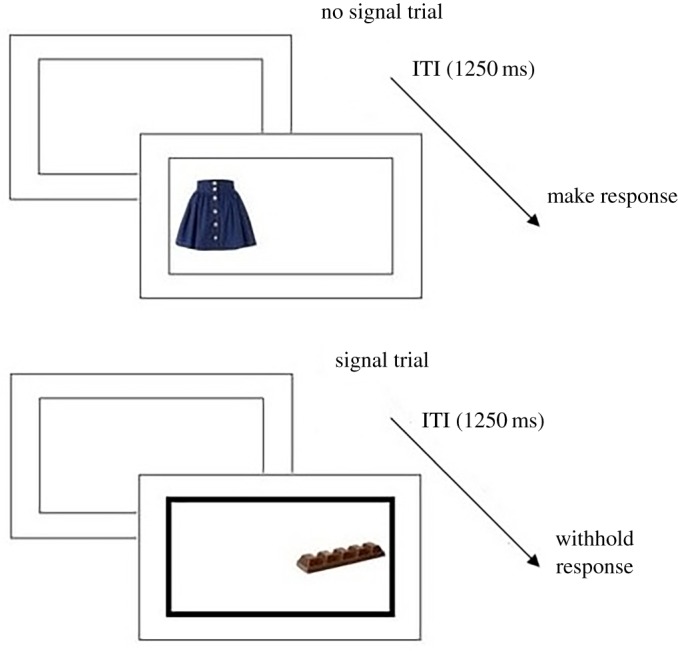
Visual schematic of the Go/No-Go training task. ITI = inter-trial interval.

The training task lasted approximately 15 min and consisted of eight blocks of 36 trials with a 15 s break between each block (see figure 1 for a visual schematic of the procedure). The blocks randomly presented nine images of unhealthy foods (three images each of chocolate, crisps and biscuits), nine images of healthy foods (three images each of fruit, rice cakes and salad vegetables) and 18 filler images (clothes; three each of jeans, shirts, jumpers, socks, skirts and ties). One stimulus of each food type was a photographed image of the corresponding food item that was presented in the snack buffet. All images were close-up views of the food item against a white background; images were carefully selected on the basis that there were no additional ingredients or packaging, and they were matched for size and complexity. Each trial began with the presentation of a central rectangle (inter-trial interval; ITI, 1250 ms). A stimulus was then presented within this rectangle randomly, and with equal probability, to either the left or right hand side. Participants were required to respond to the location of the stimulus as quickly and accurately as possible using their left and right index fingers (using the ‘C’ and ‘M’ keys, respectively). A no-go signal (the fixation rectangle turned bold for the duration of the trial) was presented on 50% of trials indicating that the participant must withhold their response for that trial. All of the unhealthy food images were presented with a signal (100% mapping), none of the healthy foods were presented with a signal (0% mapping) and half of the filler images were presented with a signal (50% mapping; see figure 2 for visual schematic). All instructions were presented electronically before the training task and read verbatim by the experimenter. All tasks were programmed in MATLAB (Mathworks, Natick, MA) using Psychophysics Toolbox (www.psychtoolbox.org) and all stimuli were presented on a 19-inch flat-panel LCD monitor. The training task was identical to that used by Adams *et al*. [33].

#### 2.2.3. Snack buffet

Ad libitum food consumption was measured using a snack buffet. A snack buffet has frequently been used in studies of food take and is considered to be a valid measure of consumption [53]. Four unhealthy (crisps, biscuits, chocolate and cheese bites) and four healthy foods (carrots, grapes, rice cakes and breadsticks) were presented in a pseudorandom order (to minimize the effect of proximity on intake; see electronic supplementary material, table S6 for weight and nutritional information). Upon entering the buffet, participants were instructed to eat as much food as they liked, but to ensure that they were not feeling hungry when the experimenter returned after 20 min (in order to replenish their glucose levels, consistent with the cover story). They were then left alone with a battery of filler questionnaires (see Questionnaires below). Unknown to the participants, all food was weighed before and after the buffet to determine calorie consumption. The snack buffet and cover story were also identical to those used by Adams *et al*. [33].

#### 2.2.4. State food craving

State food craving was measured with the General Food Craving Questionnaire – State Version (G-FCQ-S; [50]). The questionnaire consists of 15 items that measure the strength of food cravings; participants are asked to indicate how much they agree with each statement ‘at this very moment’ using a five-point scale (from 1 ‘strongly disagree’ to 5 ‘strongly agree’). There are five craving subscales including: intense desire to eat, anticipation of relief from negative states, physiological craving, preoccupation with food or lack of control over eating and anticipation of positive reinforcement. Scores can be calculated for specific subscales or a total score can be calculated (ranging from 15 to 75).

#### 2.2.5. Speeded Go/No-Go task

As a manipulation check for the effect of tDCS on inhibitory control we included a second Go/No-Go task. To avoid floor effects and improve the detection of potential improvements in inhibitory control, we modified the training task in three ways: firstly, we used a speeded version in which the ITI and stimulus presentation time was reduced to 500 ms; secondly, we reduced the percentage of no-go trials from 50% to 33.3%; thirdly, all foods were inconsistently paired with a no-go signal (33.3% mapping).^1^ The task consisted of 15 blocks of 45 trials and lasted approximately 15 min. The stimuli were the same as those presented in the training task with the addition of nine novel unhealthy foods (5 sweet, 4 savoury). Instructions were presented electronically and participants were warned verbally about the faster presentation time for this task.

#### 2.2.6. Task-relevant questionnaires

Participants completed three 100 mm visual analogue scales to measure hunger, fullness and desire to eat, and the G-FCQ-S to measure food craving [50]. We also measured mood (using the Positive and Negative Affect Schedule, PANAS; [49]) and included two questions regarding discomfort/pain and nausea to rule out differences in food consumption due to these potential effects of tDCS.

#### 2.2.7. Filler questionnaires

As in our previous studies [33,37], filler questionnaires were provided during the snack buffet to keep participants occupied for the duration of the snacking phase. The questionnaires included the Brief Self Control Scale (BSCS; [55]), the Big Five Inventory (BFI; [56]), the Emotion Regulation Questionnaire (ERQ; [57]), the UPPS impulsive behaviour scale [58], the Attentional Control Questionnaire (ACQ; [59]) and the Mood and Anxiety Symptom Questionnaire (MASQ-62; [60], identical to those used in Adams *et al.* [33] and Lawrence *et al.* [37]).

### 2.3. Procedure

At least one week prior to the study, all participants were screened for eligibility criteria and were asked to complete the Restraint Scale [61]. On the day of testing, participants were asked to eat something small three hours before the study and to then refrain from eating during this period, thus standardizing appetite and food motivation. All testing sessions therefore took place between 12 pm and 8 pm. This instruction was also consistent with the cover story that justified the snacking phase (that we were measuring the effects of glucose levels on task performance). Upon arrival, participants completed a consent form and two brain stimulation safety screening questionnaires, followed by the task-relevant questionnaires before receiving tDCS. The first five minutes of tDCS were delivered in isolation, and the remaining 15 min were delivered alongside Go/No-Go training. Following training, participants completed the task-relevant questionnaires for a second time. Participants were then taken to another room for the snack buffet and were left for 20 min with the filler-questionnaires. Finally, participants completed the speeded Go/No-Go task in the original testing room. After completion, all participants were debriefed and their awareness of the study's aims and tDCS condition was questioned (see electronic supplementary material for all debrief questions and analyses). Participants' height and weight was then recorded to calculate their body mass index (BMI; kg/m^2^).

### 2.4. Statistical analyses

Four participants were excluded in the sham group, and three in the active group based on failure to comply with task instructions (see electronic supplementary material, table S1 and electronic supplementary material for details and analysis). A further two participants (1 active, 1 sham) were excluded because they indicated knowledge of the study aim and one participant (active) was excluded after disclosing history of an eating disorder during debrief. Following exclusions there was a final sample of 172 participants: 84 in the sham condition (66 females) and 88 in the active condition (68 females). A further four participants were excluded from analysis of the speeded Go/No-Go task due to failure to comply with task instructions: 2 from the sham condition and 2 from the active condition. All exclusions were in accordance with pre-registered criteria.

Food consumption data were explored for statistical outliers to ensure that any strong food preferences did not skew the results. The data were split according to food type and tDCS condition and values that exceeded 3 s.d. from the mean were replaced with the highest non-outlier value for that food +1. This method reduces the impact of a univariate outlier while maintaining the score as the most deviant [62]. Food consumption was analysed as a function of food type and food novelty by calculating the mean calorie value for each food category (the total calories for each food category was divided by the number of foods in that category). The consumption of healthy foods was also analysed in grams to avoid potential floor effects (see electronic supplementary material).

All frequentist statistics were computed using JASP (JASP Team, 2018). Bayes factors were also calculated to interpret null findings and assess the strength of evidence [63–65]. For early research, Bayes factors greater than 6 suggest ‘substantial’ evidence for the alternative hypothesis and Bayes factors less than 0.16 indicate ‘substantial’ evidence for the null hypothesis [51]. The Bayes factor for total calorie intake was calculated using Dienes' online calculator (see electronic supplementary material for details). With no previous literature to guide an informed prior, all other Bayesian analyses were computed in JASP using the default JZS prior (*r* = 0.707; [65,66]). The JZS prior is a non-informative objective prior that minimizes assumptions regarding expected effect size. All methods and statistical analyses were pre-registered unless stated otherwise, and all study data are available online (https://osf.io/2597q/).

## 3. Deviations from protocol

We originally hypothesized that all predicted effects would be greater in those who scored highly on measures of restrained eating. However, of the 172 participants included in the analyses, only 15 participants met the cut-off score for dietary restraint (a score of 15+ on the Restraint Scale (RS); [61]) meaning that subgroup analyses could not be conducted. However, based on the recommendation of a reviewer, we analysed data with linear mixed effect models (see §4.7.).

In addition to the between-subjects factor of tDCS (active versus sham), the design in the pre-registered protocol included separate groups of participants receiving no-go training and go-only training (i.e. 2 × 2 design of active versus sham × go versus no-go). The no-go groups (active and sham tDCS) were tested first in the sequence of data collection, and in light of the null effect of tDCS reported here, the go-only groups were abandoned (no data collected). This paper thus reports the hypotheses and analysis plans for the no-go group only.

The study protocol (13 Feb 2015) is available at https://osf.io/z2xf8/. Data collection commenced on 16/02/15 and was completed on 30/01/18. The protocol was updated on 1 Aug 2016, after collection of 52 participants (but prior to the final analysis), to include fasting and food allergies as exclusion criteria https://osf.io/a5gqu/. No participants that were included in the sample prior to the protocol amendment were excluded from further analysis.

## 4. Results

### 4.1. Group differences: pre-registered

Demographic, state and trait variables were analysed to ensure there were no statistically significant differences between tDCS groups at baseline. There were no significant differences in gender ratio ($\chi_{1}^{2}=0.04$, *p* = 0.837, *ϕ* = 0.02, *B*_JZS_ = 0.16), age, BMI, dietary restraint, hunger, fullness, desire to eat, positive affect, negative affect, total craving score, craving subscales or hours since food (all *t*s < 1.5, all *p*s > 0.05, all *B*_JZS_ < 0.44; see electronic supplementary material, table S2). Within-subjects differences in hunger, fullness, desire to eat, positive affect and negative affect between pre- and post-tDCS phases were then compared between tDCS groups; no significant differences were found (all *t*s < 1.6, all *p*s > 0.05, all *B*_JZS_ < 0.53; see electronic supplementary material, table S2).

### 4.2. tDCS tolerability and blinding: pre-registered

Both active and sham stimulation were well tolerated; participants were emailed a post-monitoring form 24 h after study completion and of the 114 that completed the form, only seven participants reported a minor adverse reaction (4.9%; see electronic supplementary material, table S3). However, participants receiving active stimulation did report higher levels of pain after stimulation (*M* = 1.4; s.e. *=* 0.07) compared with those receiving sham stimulation (*M =* 1.16, s.e. *=* 0.05; *t*_170_ = 2.74, *p* = 0.007, *d* = 0.42, *B*_JZS_ = 5.08). There was no significant group difference in reported nausea after stimulation (*t*_170_ = 0.49, *p* = 0.626, *d* = 0.08; *B*_JZS_ = 0.19), nor in awareness of tDCS condition ($\chi_{2}^{2}=4.68$, *p =* 0.096, *ϕ* = 0.17, *B*_JZS_ = 0.34; see electronic supplementary material, table S4), suggesting that participants remained blind to the stimulation condition.

### 4.3. Food consumption

#### 4.3.1. Pre-registered analyses

**Figure 3.**
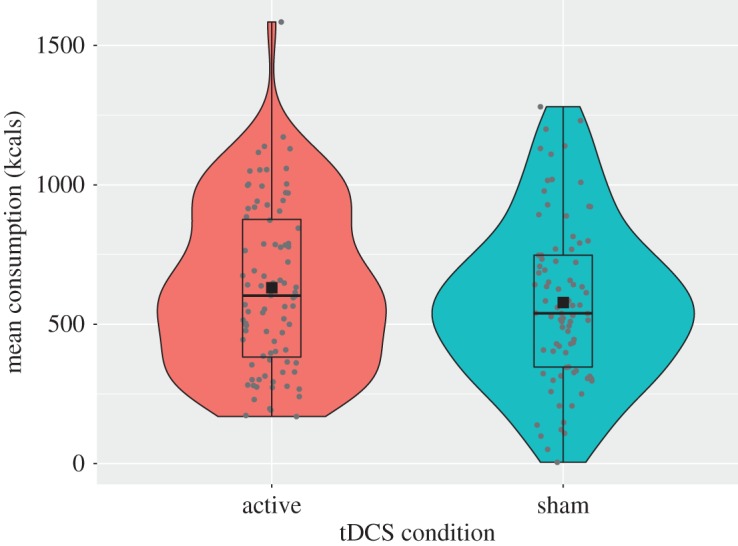
Total calorie intake as a function of tDCS condition.

A 2 × 2 × 2 mixed ANOVA (between-subjects factor: *tDCS condition* [active or sham]; within-subjects factors: *food type* [healthy or unhealthy] and *food novelty* [old or new]) revealed no significant main effect of tDCS (*F*_1,170_ = 1.54, *p* = 0.217, $\eta_{p}^{2}=0.01$; *B*_JZS_ = 0.18; addressing the primary pre-registered hypothesis). Contrary to the hypothesis, total calorie consumption was higher in the active tDCS group (*M* = 631.18, s.e. = 31.42) compared with the sham group (*M* = 577.62, s.e. = 31.78; see figure 3). A Bayesian comparison of sham and active tDCS with an informative prior (see electronic supplementary material, section 1 and section 5) revealed a Bayes factor of 0.19, indicating moderate evidence in favour of the null hypothesis (H0) over the experimental hypothesis (H1).

The mixed ANOVA also revealed no significant interaction between tDCS and food type (*F*_1,170_ = 0.87, *p* = 0.353, $\eta_{p}^{2}=0.001$; *B*_JZS_ = 0.12), or between tDCS and food novelty (*F*_1,170_ = 0.08, *p* = 0.772, $\eta_{p}^{2}<0.001$; *B*_JZS_ = 0.11). The three-way interaction between tDCS condition, food type, and food novelty was also non-significant (*F*_1,170_ = 0.004, *p* = 0.947, $\eta_{p}^{2}<0.001$; *B*_JZS_ = 0.18).

Using a multiple regression we additionally explored whether any demographic variables were significant predictors of food intake (see electronic supplementary material, table S7). BMI was a significant predictor, and as such was added as a covariate in the three pre-registered ANOVAS. The inclusion did not affect the results significantly (see electronic supplementary material for all outputs).

#### 4.3.2. Exploratory analyses

Foods were split between unhealthy and healthy and analysed separately. Participants receiving active stimulation consumed 6% more calories from unhealthy foods (active: *M* = 497.1, s.e. = 30.4; sham: *M* = 467.65, s.e. = 28.83; *t*_170_ = 0.7, *p* = 0.484, *d* = 0.11; *B*_JZS_ = 0.21 suggesting anecdotal-to-moderate evidence in favour of H0 over H1) and 22% more calories from healthy foods than those receiving sham stimulation (active: *M* = 134.07, s.e. = 8.15; sham: *M* = 109.98, s.e. = 6.7; *t*_170_ = 2.27, *p* = 0.024, *d* = 0.35; *B*_JZS_ = 1.77 suggesting anecdotal evidence that active tDCS resulted in greater consumption of healthy calories compared with sham tDCS).

To account for possible floor effects caused by the low caloric value of the healthy foods, we also analysed healthy food consumption in grams using a 2 × 2 mixed ANOVA (between-subjects factor: *tDCS condition* [active or sham]; within-subjects factor: *food novelty* [old or new]). There was a significant main effect of tDCS condition with participants in the active group consuming more healthy food compared with the sham group (*F*_1,170_ = 7.08; *p* = 0.009, $\eta_{p}^{2}=0.04$, *B*_JZS_ = 0.52). A significant interaction between tDCS condition and novelty was also observed (*F*_1,170_ = 7.37; *p* = 0.007, $\eta_{p}^{2}=0.01$, *B*_JZS_ = 7.86). Analysis of simple main effects revealed that participants consumed significantly more calories from healthy old foods in the active stimulation group (*M* = 160.1, s.e. = 11.5) compared with sham (*M* = 122.6, s.e. = 7.98; *p* = 0.008; *B*_JZS_ = 4.41), but not for healthy new foods (active: *M* = 5.4, s.e. = 0.67; sham: *M* = 5.9, s.e. = 0.69; *p* = 0.57; *B*_JZS_ = 0.19).

Furthermore, we additionally split foods by sweet and savoury to see whether food type played a role in the effectiveness of tDCS. A 2 × 2 ANOVA (between-subjects factor: *tDCS condition* [active or sham]; within-subjects factor: *food type* [sweet or savoury]) revealed a main effect of food type (*F*_1,170_ = 11.01, *p* = 0.001, $\eta_{p}^{2}=0.06$; *B*_JZS_ = 20.73) indicating that participants ate significantly more calories from sweet foods than savoury foods. However, there was no significant main effect of tDCS (*F*_1,170_ = 1.44, *p* = 0.233, $\eta_{p}^{2}=0.01$; *B*_JZS_ = 0.29) and no significant interaction between the two factors (*F*_1,170_ = 0.51, *p* = 0.478, $\eta_{p}^{2}=0.003$; *B*_JZS_ = 0.21; for details of the consumption of individual foods see electronic supplementary material, table S8).

### 4.4. Food craving: pre-registered

**Figure 4.**
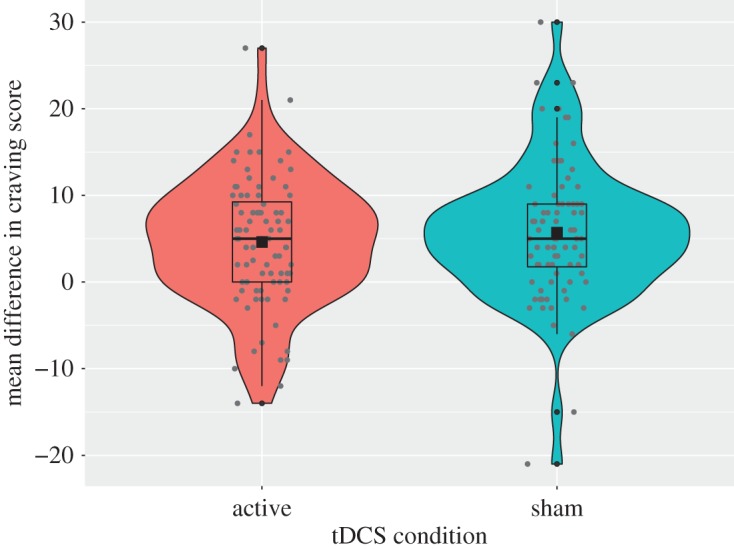
Change in state craving score as a function of tDCS condition (pre-stimulation scores were subtracted from post-stimulation scores so that a positive score indicates increased craving and a negative score would indicate decreased craving).

A 2 × 2 mixed ANOVA for total state craving scores (between-subjects factor: *tDCS condition* [active or sham]; within-subjects factor: *time* [pre- or post-stimulation]) revealed no significant effect of tDCS (*F*_1,170_ = 0.66, *p =* 0.420*,* $\eta_{p}^{2}=0.004$; *B*_JZS_ = 0.37; secondary pre-registered hypothesis; see figure 4) as well as no significant interaction between tDCS and time (*F*_1,170_ = 0.88, *p* = 0.349, $\eta_{p}^{2}=0.004$; *B*_JZS_ = 0.28).

### 4.5. Effect of tDCS on response inhibition

#### 4.5.1. Pre-registered analyses

**Figure 5.**
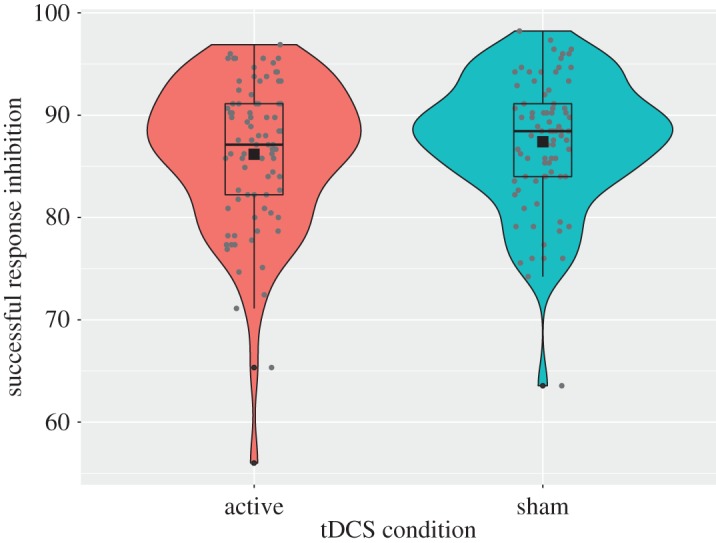
Percentage successful response inhibition in the speeded Go/No-Go task as a function of tDCS condition.

A 2 × 4 mixed ANOVA on percent of successful no-go responses in the speeded task (between-subjects factor: *tDCS condition* [active or sham]; within-subjects factor: *stimulus type* [unhealthy old, unhealthy new, healthy, filler]) revealed no significant main effect of tDCS condition (active: *M* = 86.18, s.e. = 0.8; sham: *M* = 87.4, s.e. = 0.7; *F*_1,170_ = 0.86, *p* = 0.355, $\eta_{p}^{2}=0.01$; *B*_JZS_ = 0.33; pre-registered manipulation check; see figure 5) and no statistically significant interaction between tDCS condition and stimulus type (*F*_1,170_ = 1.54, *p* = 0.205, $\eta_{p}^{2}=0.01$; *B*_JZS_ = 0.1). There was, however, a significant main effect of stimulus type (*F*_1,170_ = 12.75, *p* < 0.001, $\eta_{p}^{2}=0.07$; *B*_JZS_ = 155 512), with more successful response inhibition for unhealthy old foods (*M* = 88.13, s.e. = 0.61) than healthy foods (*M* = 85.3, s.e. = 0.69; *p* < 0.001), as well as more successful response inhibition for unhealthy new foods (*M* = 87.99, s.e. = 0.62) compared with healthy foods (*p* < 0.001) and filler items (*M* = 86.66, s.e. = 0.59; *p* = 0.04).

#### 4.5.2. Exploratory analyses

Based on the weak evidence that active tDCS resulted in greater consumption of healthy calories compared with sham tDCS (see §4.3.2), we performed an exploratory analysis to investigate whether this increase in consumption might be explained by active tDCS enhancing go-training effects to healthy foods. Descriptively, participants in the active tDCS group exhibited faster go reaction times to healthy foods (*M* = 354.06, s.e. = 2.37) compared with the sham group (*M* = 359.71, s.e. = 2.81). However a 2 × 4 mixed ANOVA on go reaction time in the speeded task (between-subjects factor: *tDCS condition* [active or sham]; within-subjects factor: *stimulus type* [unhealthy old, unhealthy new, healthy, filler]) revealed no significant main effect of tDCS (*F*_1,165_ = 3.62, *p* = 0.059, $\eta_{p}^{2}=0.02$; *B*_JZS_ = 1.35) and no significant interaction (*F*_3,495_ = 1.51, *p* = 0.211, $\eta_{p}^{2}=0.01$; *B*_JZS_ = 0.1).

### 4.6. Training data: exploratory analyses

Analyses of the data from the training task revealed that there were no significant differences between tDCS conditions for successful inhibition (*t*_170_ = 0.21, *p* = 0.833, *d* = 0.03, *B*_JZS_ = 0.17), go reaction time (*t*_170_ = 1.81, *p* = 0.073, *d* = 0.28, *B*_JZS_ = 0.74), or percentage of errors (*t*_170_ = 0.81, *p* = 0.417, *d* = 0.12, *B*_JZS_ = 0.22).

To demonstrate evidence of learning in the training task we performed a 2 × 2 mixed ANOVA on go reaction time (between-subjects factor: *tDCS condition* [active or sham]; within-subjects factor: *stimulus type* [healthy, filler]) to investigate whether participants were faster to respond to healthy foods compared to filler images. A significant main effect of stimulus type (*F*_1,170_ = 210.75, *p* < 0.001, $\eta_{p}^{2}=0.55$; *B*_JZS_ = 1.259 × 10^28^) confirmed that participants were faster at responding to healthy foods (*M* = 481.01, s.e. = 5.18) compared to filler images (*M* = 504.06, s.e. = 5.77), however we found no significant main effect of tDCS (*F*_1,170_ = 3.26, *p* = 0.073, $\eta_{p}^{2}=0.02$; *B*_JZS_ = 1.08) or interaction between tDCS and stimulus type (*F*_1,170_ = 0.87, *p* = 0.353, $\eta_{p}^{2}=0.002$; *B*_JZS_ = 0.01).

We also undertook a corresponding 2 × 2 mixed ANOVA on successful response inhibition (between-subjects factor: *tDCS condition* [active or sham]; within-subjects factor: *stimulus type* [unhealthy, filler]) to test whether, as expected by the training, participants would be better at inhibiting to unhealthy foods compared with filler images. This analysis revealed a significant main effect of stimulus type (*F*_1,170_ = 50.01, *p* < 0.001, $\eta_{p}^{2}=0.23$; *B*_JZS_ = 2.434 × 10^8^) with participants exhibiting a higher percentage of successful stopping to unhealthy foods (*M* = 96.55, s.e. = 0.23) compared with filler images (*M* = 94.54, s.e. = 0.29), but again, no significant main effect of tDCS (*F*_1,170_ = 0.04, *p* = 0.833, $\eta_{p}^{2}<0.001$; *B*_JZS_ = 0.16), or interaction between tDCS and stimulus type (*F*_1,170_ = 0.04, *p* = 0.847, $\eta_{p}^{2}<0.001$; *B*_JZS_ = 0.16).

### 4.7. Dietary restraint: exploratory analyses

As previous research has shown that inhibition training may be most effective for individuals who score higher on measures of dietary restraint, we hypothesized in our pre-registration that the effects of active tDCS and no-go training would be greatest or only present for those who met the cut-off score for restrained eating. However as only 15 of 172 participants met the criteria for dietary restraint we could not perform the analyses as defined in the pre-registered protocol. Instead we undertook a linear mixed effects analysis in R [67] using the lme4 package ([68]; between-subjects factor: *tDCS condition* [active or sham]; within-subjects factor: *time* [pre- or post-stimulation]; continuous factor: *restraint*). *p*-Values were calculated from degrees of freedom estimated using Satterthwaite's method [69]. This analysis revealed no significant main effect of tDCS (*F*_1,168_ = 1.27, *p* = 0.261), restraint (*F*_1,168_ = 0.55, *p* = 0.460) or time (*F*_1,168_ = 3.08, *p* = 0.081), and no significant interactions between tDCS and restraint (*F*_1,168_ = 0.68, *p* = 0.412), tDCS and time (*F*_1,168_ = 0.04, *p* = 0.838), time and restraint (*F*_1,168_ = 0.01, *p* = 0.920) or the three-way interaction between time, tDCS and restraint.

## 5. Discussion

The aim of this study was to investigate whether the application of tDCS to the DLPFC, alongside inhibition training, could reduce food consumption and food craving. Previous research has shown tDCS to be an effective way of reducing food cravings, although findings for food consumption are more uncertain [25,26,70,71]. Here we recruited a larger-than-typical sample size to ensure sufficient statistical power to detect moderate changes in eating behaviour, and we added a cognitive target (go/no-go training) in an attempt to boost the effectiveness of active stimulation [17,18,31,32]. Our protocol was sham-controlled and double-blind to rule out potential experimenter effects and demand characteristics; our results suggest that blinding was successful. In addition, our protocol and analyses were pre-registered to ensure transparent research practices and minimize bias. Contrary to our hypotheses, we found no evidence for an effect of active tDCS on reduced food cravings or total food consumption.

Although these results are in contrast to previous research they are consistent with a recent study that failed to show an effect of tDCS on cravings and consumption in a sample of healthy individuals ([70]; see also [72]). Furthermore, a recent meta-analysis concluded that single-session tDCS of the DLPFC was not effective at modulating food cravings and that effects on food consumption were unreliable [30]. The current literature on the effectiveness of tDCS as an intervention for eating-related behaviour therefore appears to be conflicting. One possible explanation for the difference across findings is the samples used. Previous studies showing positive results have typically recruited participants who self-identified as having strong and frequent food cravings [25,26,28] as well as cravings specific to the foods used in the experiments [24,27]. Although we selected commonly craved foods for the current study we did not make any attempt to pre-screen participants for trait food craving. Similarly, Georgii *et al*. [70] recruited an unselected sample and found no difference between active and sham stimulation for either state food cravings or desire to eat the foods. Studies that have found positive effects of tDCS without selecting individuals based on trait craving have either included obese samples or have used repeated sessions of tDCS [71,73,74]; with a sample of healthy men Jauch-Chara *et al.* [74] only reported a significant reduction in food intake after eight daily stimulation sessions, with no effects after a single session. Furthermore, the results reported by Ray *et al.* [71] were also dependent on gender, restrained eating and different facets of impulsivity; a reduction in craving was specific to women with low attentional impulsivity and a reduction in food consumption was only found for men who were either low in restrained eating or high in non-planning impulsivity. With a very small sample size (eight females, 10 males) and lack of explicit bias control (pre-registration) it is difficult to draw firm conclusions from this study alone, yet together the current literature indicates a need to consider the role of cognitive traits and individual differences in this line of research.

Studies have also reported specific effects of tDCS according to different macronutrients, which may explain why we did not see positive results for a general food craving measure such as the G-FCQ-S [50]. However, these results again appear variable across the literature. For example, Goldman *et al*. [25] reported a reduction in craving for carbohydrates following active stimulation whereas two subsequent studies found no effects for carbohydrate craving [28,29]. Likewise, Jauch-Chara *et al*. [74] reported that the effect of repeated tDCS on total calorie consumption was mainly due to a reduction in carbohydrate intake, although Gluck *et al*. [73] reported no effect on carbohydrate intake following three sessions of anodal compared to cathodal stimulation. Contrasting results have also been reported for fats [25,28,73] and protein [29,73]; however, the one taste category that does appear to be consistently associated with reduced craving and consumption is sweet foods [25,26,28,29,73].

Sweet foods, more specifically foods high in sugar, are often thought of as having addictive potential with reward often considered a key driver for consumption in the absence of hunger. Neurotransmitters have been shown to modulate food intake, particularly for specific macronutrients, for example consumption of sugar releases dopamine in the same way as consumption of addictive substances, and the behavioural effects of sugar consumption and substance use are similar [75,76]. In addition to dopamine, research also indicates the importance of endogenous opioids in the preference of high-sugar foods [77]. Cravings for sweet foods are also more common compared to those for savoury foods which may explain why effects of tDCS are more consistent for such foods [78]. Exploratory analyses in the current study indicated that participants consumed significantly more calories from sweet foods, however, we found no interaction between food type and tDCS condition.

As well as exploring differences across macronutrients, another approach is to consider personal preferences. By asking participants to rank their favourite of the foods presented, both Burgess *et al*. [29] and Ray *et al*. [71] were able to demonstrate that effects of active stimulation on food consumption were specific to preferred foods.

Taken together, this literature indicates that the utilization of tDCS as a potential intervention for eating-related behaviour is preliminary. There remain questions with regards to who can benefit the most from such an intervention and under what circumstances. Lessons and future directions can be taken from similar interventions such as food-related cognitive control training, which has been shown to be most effective for those who are high in impulsivity and restrained eating [36,40]. There is already some evidence in the tDCS literature to suggest that cognitive traits may play a significant role in determining the direction of effects [26,71]; however, replication with larger sample sizes is necessary to verify such claims. The investigation of individual differences may also help us to understand more about the underlying mechanisms; for example tDCS may be more effective for those who are impulsive due to hypoactivity in prefrontal areas related to inhibitory control. In the current study, we explored whether stimulation of the DLPFC during inhibition training would result in improved inhibitory performance (see [48]). Our results revealed no significant differences between active and sham stimulation during training but provided weak evidence that active stimulation may speed reaction times without increasing commission errors in a later task. Lapenta *et al*. [27] also investigated whether the effect of DLPFC stimulation on reduced craving was due to modulation of inhibitory control. They found that tDCS resulted in a significant increase in the frontal P3a component suggesting enhanced inhibition of an overt response; however, they found no significant differences in behavioural performance between active and sham conditions, which could have been due to ceiling effects.

Another possibility is that stimulation of the DLPFC serves to reduce the hedonic value of food. Hare *et al.* [79] showed that increased activity in the DLPFC was associated with successful self-control when making food choices and was also found to downregulate the goal value of unhealthy palatable foods. It is possible therefore that tDCS effects could be stronger for those who demonstrate high reward sensitivity and who find food particularly rewarding. As discussed above, early evidence for this proposal comes from positive effects with samples of individuals who score highly on trait food craving [24,25,27]. Moreover, inhibition training studies have proposed that devaluation of food stimuli may be a potential mediator for the effect of training on behaviour [80]. However it should be noted that recent research has shown that devaluation as a result of inhibitory control training correlates with activity in brain regions other than the DLPFC (e.g. [81]).

An exploratory analysis indicated that active prefrontal tDCS may cause increased consumption of healthy foods in comparison to sham tDCS, raising the question of whether this tDCS protocol could have the potential to enhance consumption when used alongside go-training such as cue-approach training (CAT). Whereas inhibitory control training is used with the aim of reducing food consumption, CAT aims to increase consumption for pre-determined foods. For example, Kakoschke *et al.* [82] used a visual dot probe task to investigate whether training attention towards foods could modify food consumption. They found that when participants were trained to attend toward healthy foods and away from unhealthy foods, they consumed significantly more healthy food in a subsequent taste test in comparison to a group that had trained to orient attention toward unhealthy foods and away from healthy foods. Combining tDCS with CAT may be a promising route to explore the increase in consumption of healthy, minimally processed foods, which could potentially reduce consumption of more heavily processed foods.

An additional avenue for future investigation is to consider improvements in brain stimulation techniques as well as comparison between different methodologies. Transcranial magnetic stimulation (TMS) is another method that has been used to modulate food cravings and consumption (e.g. [83,84]). In the same meta-analysis that indicated no effect of tDCS on food craving, Lowe *et al*. [30] found a significant effect of TMS in the reduction of food cravings. In addition to producing stronger stimulation of cortex [85], TMS also has greater spatial focality than tDCS depending on the type of coil [86]. Conventional tDCS involves the delivery of a current via 2 electrodes, typically quite large in size. Electrical field modelling of the current flow indicates that large areas of the cortex can be disrupted during stimulation leading to concerns regarding focality [87]. These concerns have fuelled developments in tDCS techniques and the production of a more focal application. High definition (HD) tDCS involves the use of much smaller electrodes (typically 1 cm) placed in a 4 × 1 montage, with one single electrode placed over the region of interest, and the remaining four arranged in a ring around the outside of the central electrode, resulting in a smaller area of stimulation [88]. Few studies have yet compared the effects of the two techniques, but preliminary research has found the duration of effects to be improved with HD-tDCS [89]. If the negative findings we observed are due to lack of focality then HD-tDCS may provide a fruitful next step, as may the application of multiple sessions (e.g. [74]).^2^

In summary, the current study failed to replicate the effects of tDCS on reduced food craving or food consumption within a larger-than-usual sample. While there may be potential for tDCS as an intervention for unhealthy eating behaviour, our findings and those of Lowe *et al*. [30] highlight the need for such studies to include larger sample sizes and explicit bias control (including study pre-registration), thus allowing for more robust and transparent insights (see also [90]). After a surge in papers reporting the effectiveness of tDCS in the last decade, the effects have also been questioned more recently with reference to individual differences [91] and the efficacy of single-sessions [92]. Future research should be guided by these findings to focus on the importance of optimal study design as well as the potential role of individual differences.

## Supplementary Material

Supplementary Analyses

## Footnotes

1 The commission error rate on the training task is typically very low (approx. 5%) making it difficult to detect any potential improvements in inhibitory control. It was believed that these changes would encourage rapid responding, which, as a result of the speed-accuracy trade-off would also increase the rate of commission errors (see [54]). A pilot study (*N* = 13) we conducted with non-food images (e.g. household items including electrical goods and furniture) showed this to be the case with a mean commission error rate of 24.44%, s.e. = 3.53%.

2 We also cannot rule out the possibility that inhibition training might counteract the effects of tDCS, should the two interventions in any way compete to diminish the sensitivity of the DLPFC to stimulation. This remains to be tested.

## Data accessibility

The pre-registered study protocol, anonymised study data and JASP outputs are available on the Open Science Framework (https://osf.io/2597q/).
