# Supplementary material for: Prefrontal brain stimulation during food-related inhibition training: effects on food craving, food consumption and inhibitory control
Source: R Soc Open Sci. 2019 Jan 9;6(1):181186. doi: 10.1098/rsos.181186 (PMC6366210; doi:10.1098/rsos.181186)
Supplement: Supplementary Analyses [file rsos181186supp1.docx]

**Supplementary Information:**

**Prefrontal brain stimulation during food-related inhibition training: Effects on food craving, food consumption and inhibitory control**

**1. Bayesian Stopping Rule**

In our pre-registered protocol we aimed to determine our sample size using a Bayesian inferential stopping rule for the main effect of total calorie intake between tDCS groups^[[1]](#footnote-1)^. We initially collected data for 20 participants and then planned to continue with data collection until the Bayes factor provided substantial evidence for either the experimental hypothesis (H1; B>3) or the null hypothesis (H0; B<0.33). Based on recent recommendations for early research we adjusted our threshold to achieve a Bayes factor of either >6 (substantial evidence for H1) or <0.16 (substantial evidence for H0; Schönbrodt et al., 2017). Using Bayesian hypothesis testing allows for a flexible stopping rule on data collection without correcting for the elevation of Type I error, as would be required under a conventional frequentist approach (Dienes, 2011, 2014).

To calculate an expected difference for total calorie intake between groups (the Bayesian prior), we used data available at the time of the pre-registered protocol. At the time, the only publication reporting an effect of tDCS on calorie consumption was Fregni et al. (2008) who showed a difference of ~110 calories between sham and anodal right/ cathodal left tDCS of DLPFC^[[2]](#footnote-2)^. Following Dienes (2011) we applied a half-normal distribution with a mean value of 0 and a standard deviation that corresponded to the expected difference. For the sample mean and standard error, a between-subjects t-test was performed for the effect of tDCS condition (active or sham) on total food intake. The mean difference and standard error of the difference for this comparison were entered into Dienes’ online calculator^[[3]](#footnote-3)^.

**2. Participant Debrief**

At the end of the study, participants were asked a series of questions. To probe for awareness of the study aims, participants were asked 1) whether they were aware of the aim of the study, 2) whether they noticed anything in particular about the training task, 3) whether they thought the signals were distributed evenly, randomly or grouped, and 4) whether their performance on the training task had any influence on the questionnaires, the snack buffet, or speeded Go/No-Go task. They were then asked if they had participated in any related studies, if they were currently dieting, if they had any history of eating disorders, and at what time they last ate to allow for exclusions based on these criteria. Checks for these factors were made prior to testing but were asked again during the debrief for clarification.

**3. Outliers & Exclusions** (see Supplementary Table 1).

*Amendment*

An amendment was made to the pre-registration on 01/08/2016 to exclude any participants who were currently fasting. At the time of the amendment 52 participants had taken part.

*Training task*

Training data was checked for the mean reaction time and percentage of incorrect/ missed responses on no-signal trials and the commission error rate for signal-trials to allow for exclusions based on failure to comply with task instructions. Data was split according to tDCS condition and participants were excluded if their mean reaction time on no-signal trials exceeded 3SDs from the group mean, if they had an error rate of >15% for no-signal trials, or if their commission error rate for signal trials exceeded 3SDs from the group mean. Four participants were excluded in the sham group, and three in the active group based on these criteria.

*Debrief*

Although no participants explicitly guessed the aim of the study, two participants were excluded based on the comments made consistent with our pre-registered protocol. One participant asked if the food was weighed after the buffet, and one implied that the Go/No-Go training was designed to make the participant like healthy foods.

*Speeded Go/No-Go task*

Exclusion criteria for the speeded *Go/No-Go* task was consistent with that of the training task. Data was split according to tDCS condition and outliers were identified; exclusions were based on the same criteria as the *Go/No-Go* training data for mean reaction time and commission error rate for signal trials (>3SDs from the mean). However, to address the added difficulty of the speeded *Go/No-Go* task, errors for no-signal trials were also based on group means and participants were excluded if their error rate exceeded 3SDs. Two participants from each tDCS condition were excluded from the response inhibition analyses based on these criteria.

**Supplementary Table 1.** Reasons for participant exclusions.

|  | Condition | |
| --- | --- | --- |
| Reason for exclusion | Sham | Active |
| Go/No-Go Training |  |  |
| Mean RT (>3SDs group mean) | 1 | 0 |
| % no-signal errors (>15%) | 1 | 0 |
| Commission errors (>3SDs group mean) | 2 | 3 |
| Speeded Go/No-Go Task |  |  |
| Mean RT (>3SDs group mean) | 0 | 0 |
| % no-signal errors (>3SDs group mean) | 2 | 1 |
| Commission errors (>3SDs group mean) | 0 | 2 |
| Dieting or eating disorder | 0 | 1 |
| Debrief comments | 1 | 1 |

Note. RT = reaction time. Exclusions for performance on the speeded Go/No-Go task were only excluded from response inhibition analyses

**Supplementary Table 2.** Group characteristics and between-group significance tests (SE within parentheses).

|  | Sham | Active |  |  |  |  |
| --- | --- | --- | --- | --- | --- | --- |
|  | (n=84) | (n=88) | *t*= | *p*= | *d*= | *B_JZS_* |
| Gender (% female) | 79% | 77% |  |  |  |  |
| Age | 21.1 (0.45) | 20.51 (0.28) | 1.13 | 0.261 | 0.17 | 0.3 |
| BMI | 22.52 (0.38) | 23.24 (0.41) | 1.28 | 0.203 | 0.2 | 0.35 |
| RS | 7.82 (0.49) | 8.09 (0.49) | 0.39 | 0.699 | 0.06 | 0.18 |
| Hours since food | 5.87 (0.51) | 6.09 (0.5) | 0.31 | 0.758 | 0.05 | 0.17 |
| Hunger (baseline) | 5.04 (0.21) | 5.19 (0.19) | 0.51 | 0.609 | 0.08 | 0.19 |
| Hunger (diff post-pre) | -1.47 (0.16) | -1.23 (0.15) | 1.07 | 0.287 | 0.16 | 0.28 |
| Fullness (baseline) | 1.38 (0.17) | 1.64 (0.16) | 1.16 | 0.249 | 0.18 | 0.31 |
| Fullness (diff post-pre) | -0.35 (0.1) | -0.52 (0.12) | 1.16 | 0.248 | 0.18 | 0.31 |
| Desire to eat (baseline) | 5.47 (0.25) | 5.6 (0.23) | 0.39 | 0.699 | 0.06 | 0.18 |
| Desire to eat (diff post-pre) | -1.44 (0.19) | -1.23 (0.16) | 0.85 | 0.399 | 0.13 | 0.23 |
| Positive affect (baseline) | 28.45 (0.64) | 27.17 (0.71) | 1.34 | 0.183 | 0.21 | 0.38 |
| Positive affect (diff post-pre) | -3.43 (0.55) | -4.66 (0.54) | 1.58 | 0.116 | 0.24 | 0.52 |
| Negative affect (baseline) | 12.84 (0.32) | 12.2 (0.31) | 1.44 | 0.152 | 0.22 | 0.43 |
| Negative affect (diff post-pre) | -1.11 (0.29) | -0.5 (0.28) | 1.51 | 0.134 | 0.23 | 0.47 |
| G-FCQ-S (baseline) | 46.33 (1.31) | 45.41 (1.3) | 0.5 | 0.616 | 0.08 | 0.19 |
| G-FCQ-S (diff post-pre) | 5.7 (0.85) | 4.62 (0.78) | 0.94 | 0.349 | 0.14 | 0.25 |
| G-FCQ-S_DE (baseline) | 9.98 (0.36) | 10 (0.32) | 0.05 | 0.961 | 0.01 | 0.17 |
| G-FCQ-S_PR (baseline) | 9.23 (0.36) | 9.08 (0.3) | 0.32 | 0.751 | 0.05 | 0.17 |
| G-FCQ-S_NR (baseline) | 9.95 (0.3) | 9.78 (0.29) | 0.4 | 0.690 | 0.06 | 0.18 |
| G-FCQ-S_OP (baseline) | 7.02 (0.29) | 6.78 (0.3) | 0.58 | 0.564 | 0.09 | 0.19 |
| G-FCQ-S_PS (baseline) | 10.16 (0.3) | 9.76 (0.31) | 0.92 | 0.362 | 0.14 | 0.24 |

Note. SE = standard error; BMI = body mass index; RS = Restraint Scale; G-FCQ-S = General Food Craving Questionnaire – State Version; G-FCQ-S_DE = desire to eat; G-FCQ-S_PR = anticipation to positive reinforcement; G-FCQ-S_NR = anticipation to negative reinforcement; G-FCQ-S_OP = obsessive preoccupation; G-FCQ-S_PS = craving as a physiological state.

**Supplementary Table 3.** Incidence rates of adverse reactions reported on post-monitoring forms

|  | Sham (n=57) | Active (n=57) |
| --- | --- | --- |
| Seizure | 0 | 0 |
| Fainting/ collapse | 0 | 0 |
| Dizziness | 2 | 0 |
| Nausea/ vomiting | 1 | 0 |
| Headache | 2 | 1 |
| Muscular aches | 1 | 0 |
| Muscle spasm/ twitch | 0 | 1 |
| Insomnia | 2 | 1 |
| Sensory problems | 0 | 1 |
| Difficulty speaking/ understanding speech | 0 | 0 |
| Lack of co-ordination | 0 | 0 |
| Slowness/ impairment of thought | 1 | 1 |
| Skin irritation | 1 | 0 |
| Other | 1 | 0 |

Note. Total number of adverse reactions per tDCS condition is greater than the number of participants who reported an adverse reaction due to several participants selecting multiple reactions on the post-monitoring form.

**Supplementary Table 4.** tDCS blinding (percentages within parentheses)

|  |  | tDCS Condition | |
| --- | --- | --- | --- |
|  |  | Sham  (n=84) | Active  (n=88) |
| tDCS Guess | Sham | 32 (38.1%) | 26 (29.55%) |
|  | Active | 43 (51.19%) | 58 (65.91%) |
|  | Unsure | 9 (10.71%) | 4 (4.55%) |

**Supplementary Table 5.** Food consumption data in kCal for tDCS condition (SE within parentheses).

|  |  | Sham | Active | *t=* | *p=* | *d=* | *B_JZS_* |
| --- | --- | --- | --- | --- | --- | --- | --- |
| Total |  | 577.62 (31.78) | 631.18 (31.42) | 1.2 | 0.12 | 0.18 | 0.32 |
| Means: |  |  |  |  |  |  |  |
| Unhealthy | All | 467.64 (28.83) | 497.1 (30.4) | 0.7 | 0.484 | 0.11 | 0.207 |
|  | Old | 119.38 (8.39) | 121.83 (8.13) | 0.21 | 0.835 | 0.03 | 0.168 |
|  | New | 109.5 (11.31) | 131.62 (13.48) | 1.25 | 0.212 | 0.19 | 0.341 |
| Healthy | All | 109.98 (6.7) | 134.07 (8.15) | 2.27 | 0.024 | 0.35 | 1.774 |
|  | Old | 28.66 (1.83) | 37.51 (2.71) | 2.68 | 0.008 | 0.41 | 4.405 |
|  | New | 24 (3.35) | 21.55 (2.72) | 0.57 | 0.570 | 0.09 | 0.192 |

Note. SE = standard error; kCal = calories.

**Supplementary Table 6.** Nutritional information and weights for the foods presented in the snack buffet.

|  |  | Weight provided (g) | kCals per 100g | Fat per 100g |
| --- | --- | --- | --- | --- |
| Healthy foods | Rice cakes (mini)  *Boots organic plain rice cakes* | ~57 | 388 | 3 |
|  | Carrot batons  *Pre-cut carrots* | ~279 | 42 | 0.3 |
|  | Grapes  *Green grapes* | ~387 | 70 | 0.1 |
| Unhealthy foods | Crisps  *Tesco’s ready salted crisps* | ~76 | 550 | 36.3 |
|  | Chocolate  *Cadbury ‘Bitsa Wispa’* | ~269 | 554 | 34.2 |
|  | Biscuits (mini)  *Fox’s mini malted milk biscuits** | ~158 | 484 | 21.4 |
| Novel healthy food | Breadsticks (mini)  *Sainsbury’s mini breadsticks* | ~110 | 404 | 7.4 |
| Novel unhealthy food | Cheese bites  *ASDA’s cheese bites** | ~172 | 536 | 29.2 |

Note. Similar products were used where other products were discontinued; kCals = calories

**4. Additional Analyses**

*Predictors of food consumption*

Consistent with our pre-registered analyses we also explored whether any of the following variables were associated with total food consumption: age, BMI, RS, hours since food, hunger, fullness, desire to eat, positive affect, negative affect and baseline G-FCQ-S score. BMI was the only variable found to be significantly associated with food consumption (see Supplementary Table 7). In our pre-registration we stated that any significant variables would be included as covariates in our primary ANOVAs.

*Food Consumption*

A 2x2x2 mixed ANCOVA (between-subjects factor: *tDCS condition* [active or sham]; within-subjects factors: *food type* [healthy or unhealthy] and *food novelty* [old or new]; covariate: BMI) revealed no significant main effects of tDCS (*F*(1,168)=0.98, *p*=0.323, *ƞ*_p_²=0.01; B_JZS_=0.17), food novelty (*F*(1,168)=0.20, *p*0.652, *ƞ*_p_²=0.001; B_JZS_=1.304e+27) or BMI (*F*(1,168)=2.93, *p*<0.089, *ƞ*_p_²=0.02; B_JZS_=0.36). The interactions between food type and tDCS (*F*(1,168)=0.56, *p*=0.456, *ƞ*_p_²=0.003; B_JZS_=0.11), food novelty and tDCS (*F*(1,168)=0.004, *p*=0.952, *ƞ*_p_²<0.001; B_JZS_=0.11), and food type and food novelty (*F*(1,168)=3.11, *p*=0.080, *ƞ*_p_²=0.02; B_JZS_=5.43) were also non-significant, as was the three-way interactions between food type, food novelty and tDCS (*F*(1,168)=0.05, *p*=0.828, *ƞ*_p_²<0.001; B_JZS_=0.19). There was a significant main effect of food type (*F*(1,168)=9.81, *p*=0.002, *ƞ*_p_²=0.06; B_JZS_=1.438e+20).

*Food Craving*

A 2x2 mixed ANCOVA for total state craving scores (between-subjects factor: *tDCS condition* [active or sham]; within-subjects factor: *time* [pre or post stimulation]; covariate: BMI) revealed no main effect of tDCS (*F*(1,168)=1.31, *p*=0.254, *ƞ*_p_²=0.01; B_JZS_=0.48), time (*F*(1,168)=0.17, *p*=0.683, *ƞ*_p_²=0.001; B_JZS_=6.813e+12), or BMI (*F*(1,168)=0.16, *p*=0.691, *ƞ*_p_²=0.001; B_JZS_=0.3). Furthermore there was no significant interaction between time and tDCS (*F*(1,168)=1.18, *p*=0.278, *ƞ*_p_²=0.007; B_JZS_=0.24).

*Response Inhibition*

A 2x4 mixed ANCOVA on percent of successful no-go responses in the speeded task (between-subjects factor: *tDCS condition* [active or sham]; within-subjects factor: *stimulus type* [unhealthy old, unhealthy new, healthy, filler]; covariate: BMI) revealed no main effect of tDCS (*F*(1,163)=0.79, *p*=0.377, *ƞ*_p_²=0.01; B_JZS_=0.36), stimulus type (*F*(1,163)=1.12, *p*=0.339, *ƞ*_p_²=0.01; B_JZS_=248301.9), or BMI (*F*(1,163)=1.1, *p*=0.296, *ƞ*_p_²=0.01; B_JZS_=0.4). The interaction between stimulus type and tDCS (*F*(1,163)=1.63, *p*=0.182, *ƞ*_p_²=0.01; B_JZS_=1.18 was also non-significant).

**Supplementary Table 7.** Relationship between group characteristics and total food consumption.

|  | *p=* |
| --- | --- |
| Age | 0.54 |
| BMI | 0.02 |
| RS | 0.09 |
| Hours since food | 0.75 |
| Hunger | 0.41 |
| Fullness | 0.64 |
| Desire to eat | 0.33 |
| Positive affect | 0.31 |
| Negative affect | 0.61 |
| G-FCQ-S (baseline) | 0.31 |

Note. Model R^2^=0.13, *F*(10,160)=2.36,*p*=0.013

BMI = body mass index; RS = Restraint Scale; G-FCQ-S = General Food Craving Questionnaire – State Version

*Sweet versus savoury foods*

Some of the literature investigating the effects of tDCS on food craving and food consumption has indicated a possible influence of food type. For example, Kekic et al., (2014) found a significant effect of tDCS on craving in sweet foods, but this result was not replicated in savoury foods.

In addition to the sweet vs savoury analysis, we further split the foods and conducted a 2x2 ANOVA (between-subjects factor: *tDCS condition* [active or sham]; within-subjects factor: *food type* [unhealthy sweet and unhealthy savoury]). This revealed no main effect of food type (*F*(1,170)=2.35, *p*=0.127, *ƞ*_p_²=0.01; B_JZS_=0.38) or tDCS (*F*(1,170=0.49, *p*=0.484, *ƞ*_p_²=0.003; B_JZS_=0.19), and no interaction between the two (*F*(1,170)=2.31, *p*=0.130, *ƞ*_p_²=0.01; B_JZS_=0.49).

*Individual foods*

To explore whether there were any significant differences between individual foods, we analysed each food from the buffet individually. The only food item that resulted in a significant difference between groups was grapes, with participants who received active stimulation consuming significantly more than those who received sham stimulation (see Supplementary Table 8).

**Supplementary Table 8.** Food consumption data for individual food items in kCal for tDCS condition (SE within parentheses).

|  |  | Sham | Active | *t=* | *p=* | *d=* | *B_JZS_* |
| --- | --- | --- | --- | --- | --- | --- | --- |
| Chocolate |  | 182.12 (16) | 174.48 (14.78) | 0.35 | 0.726 | 0.05 | 0.18 |
| Carrots |  | 12.18 (1.35) | 13.18 (1.51) | 0.49 | 0.623 | 0.08 | 0.19 |
| Cheese bites |  | 109.5 (11.31) | 131.63 (13.48) | 1.25 | 0.211 | 0.19 | 0.34 |
| Bread sticks |  | 24 (3.35) | 21.55 (2.72) | 0.57 | 0.570 | 0.09 | 0.19 |
| Biscuits |  | 76.42 (9.14) | 74.18 (9.41) | 0.17 | 0.865 | 0.03 | 0.17 |
| Grapes |  | 63.71 (5.07) | 88.57 (6.75) | 2.92 | 0.004 | 0.45 | 8.18 |
| Crisps |  | 99.61 (8.99) | 116.82 (9.81) | 1.29 | 0.199 | 0.2 | 0.36 |
| Rice cakes |  | 10.09 (1.25) | 10.78 (1.4) | 0.37 | 0.716 | 0.06 | 0.18 |

Note. SE = standard error; kCal = calories.

*Debrief*

After completion of the study, participants were probed for awareness of the stimulus mappings during the training task. They were considered aware if any comments were made regarding unhealthy foods being associated with signals. Overall awareness was quite low with only 9.88% of participants noticing anything specific about the task. Within conditions, 14.29% of those in the sham group were aware, and 5.68% in the active group. A chi-square test revealed no significant difference between groups (χ ^2^(1)=3.57, *p*=0.059, *ϕ*=0.14; B_JZS_=0.65).

Participants were also asked whether they believed the training had any influence on the questionnaires, snack buffet, or the speeded Go/No-Go task. Across both groups 35.47% reported an influence; 39.29% in the sham condition and 31.82% in the active condition (χ^2^(1)=1.05, *p*=0.306, *ϕ*=0.08; B_JZS_=0.3). Influence was then split into 3 categories: task, hunger, and mood. Task related to any comments participants made regarding the training having improved their performance on the speeded Go/No-Go task due to, for example, practice effects. Hunger related to any comments about increased appetite or consumption after the training task, and mood related any comments regarding motivation, alertness or fatigue. 13.37% reported a positive effect of training on speeded performance, 19.77% reported an effect of training on increased hunger, and 1.16% reported an effect of mood. A chi-square test was conducted for each category but was not significant for task (χ ^2^(1)=1.54, *p*=0.215, *ϕ*=0.1; B_JZS_=0.27), hunger (χ ^2^(1)=0.02, *p*=0.880, *ϕ*=0.01; B_JZS_=0.15), or mood (χ ^2^(1)=1.93, *p*=0.165, *ϕ*=0.11; B_JZS_=0.09).

**5. Output of Bayesian comparison of total calories consumed in sham vs. active tDCS conditions using an informative prior (as described in Section 1 and see** [**http://www.lifesci.sussex.ac.uk/home/Zoltan_Dienes/inference/bayes_factor.swf**](http://www.lifesci.sussex.ac.uk/home/Zoltan_Dienes/inference/bayes_factor.swf)**)**


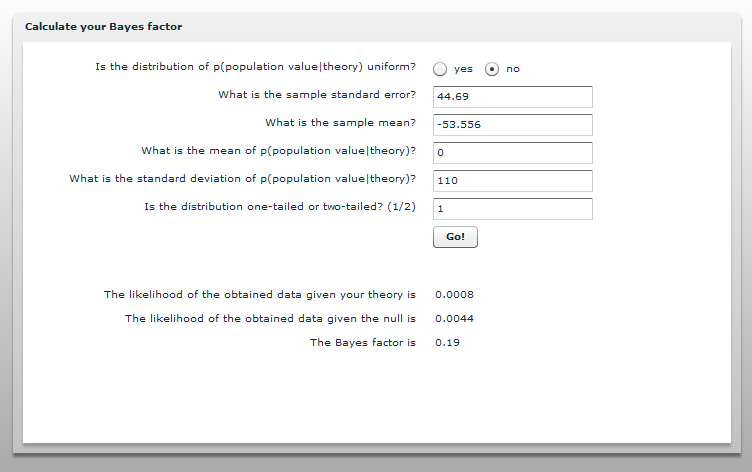


**6. Full ANOVA outputs**

Despite analyses indicating no main effects of, or interactions with tDCS, here we report all main effects and interactions not included in the manuscript.

*Food consumption (4.3)*

*Pre-registered analyses (4.3.1)*

A 2x2x2 mixed ANOVA (between-subjects factor: *tDCS condition* [active or sham]; within-subjects factors: *food type* [healthy or unhealthy] and *food novelty* [old or new]) revealed significant main effects of food type and food novelty indicating that participants ate significantly more calories from unhealthy food than healthy food (*F*(1,170)=642.4, *p<*0.001, *ƞ*_p_²=0.79; B_JZS_=1.636e+20), and significantly more calories from old foods than novel foods ( *F*(1,170)=217.93, *p*<0.001, *ƞ*_p_²=0.56; B_JZS_=1.818e+27; see Supplementary Table 5 for food consumption data). There was also a significant interaction between food type and food novelty (*F*(1,170)=263.61, *p*<0.001, *ƞ*_p_²=0.61; B_JZS_=5.28), with pairwise comparisons indicating a significant difference between old and new foods for healthy (*p*<0.001) but not unhealthy foods (*p*=0.31).

*Exploratory analyses (4.3.2)*

A 2x2 mixed ANOVA for healthy food consumption in grams (between-subjects factor: *tDCS condition* [active or sham]; within-subjects factor: *food novelty* [old or new]) revealed a main effect of novelty (*F*(1,170)=364.81; *p*<0.001, *ƞ*_p_²=0.67; B_JZS_=8.993e+54) with greater consumption of old foods compared to new foods..

*Food craving (4.4)*

A 2x2 mixed ANOVA for total state craving scores (between-subjects factor: *tDCS condition* [active or sham]; within-subjects factor: *time* [pre or post stimulation]) revealed a significant main effect of time with craving scores increasing after exposure to food images in the training task (*F*(1,170)=79.96, *p*<0.001, *ƞ*_p_²=0.32; B_JZS_=6.753e+12).

1. <https://osf.io/2597q/> [↑](#footnote-ref-1)
2. Since publishing our pre-registered protocol, Lapenta et al. (2014) reported a reduction in calorie intake following active tDCS (anodal right/ cathodal left tDCS of DLPFC) and we are currently awaiting figures from the authors. [↑](#footnote-ref-2)
3. http://www.lifesci.sussex.ac.uk/home/Zoltan_Dienes/inference/bayes_factor.swf [↑](#footnote-ref-3)
